# Application of a High-Performance, Low-Cost Portable NDIR Sensor Monitoring System for Continuous Measurements of In Situ Soil CO_2_ Fluxes

**DOI:** 10.3390/s26030761

**Published:** 2026-01-23

**Authors:** Xinyuan Zeng, Xiaoyan Chen, Lee Heng, Suarau Odutola Oshunsanya, Hanqing Yu

**Affiliations:** 1 Institute of Environment and Sustainable Development in Agriculture, Chinese Academy of Agricultural Sciences (CAAS), Haidian District, Beijing 100081, China; 82101235300@caas.cn (X.Z.); xiaoyanchen@zju.edu.cn (X.C.); 2 International Atomic Energy Agency, A-1400 Vienna, Austria; henglk2014@gmail.com; 3 Department of Soil Resources Management, University of Ibadan, Ibadan 200005, Nigeria; so.oshunsanya@ui.edu.ng

**Keywords:** soil CO_2_ monitoring, soil respiration, continuous monitoring, in situ monitoring

## Abstract

Monitoring soil CO_2_ is essential for accurately quantifying the sources and sinks of atmospheric greenhouse gases and for providing carbon emission reduction strategies. However, the limited portability and high cost of conventional soil CO_2_ monitoring equipment have severely restricted large-scale and long-term field observations. To address these constraints, this study has successfully designed and fabricated a portable and low-cost soil respiration system (SRS) based on non-dispersive infrared (NDIR) sensor technology and Long-range radio (LoRa) wireless communication. The SRS enables multi-point synchronous measurements and remote data transmission. Its reliability was rigorously evaluated through both simulated and field comparative experiments against the LI-8100A. The results demonstrated a high level of agreement between the measurements of the SRS and the LI-8100A, with the coefficients of determination (R^2^) of 0.996 and 0.997, respectively, for the simulation and field experiments, with the corresponding root mean square error (RMSE) of 0.090 and 0.089 μmol·m^−2^·s^−1^. The Bland–Altman analysis further confirmed the consistency between the two systems, with over 95% of the data points falling within the acceptable limits of agreement. These findings indicate that the self-developed SRS substantially reduces costs while maintaining reliable measurement accuracy. With its wireless transmission and multi-point deployment capabilities, the SRS offered an efficient and practical solution for addressing the challenges of monitoring spatial heterogeneity of soil respiration, demonstrating considerable potential for broader application in CO_2_ flux monitoring research.

## 1. Introduction

Soil respiration, as a key process in the carbon cycle of terrestrial ecosystems, represents the primary in which soil releases CO_2_ into the atmosphere [1]. As the dominant greenhouse gas, CO_2_ contributes most significantly to the greenhouse effect [2]. Even minor variations in soil respiration can lead to substantial changes in atmospheric CO_2_ concentrations, which in turn have a profound influence on global climate change [3]. Consequently, accurate monitoring of soil CO_2_ flux is essential to accurately quantify the sources and sinks of atmospheric greenhouse gas, and for advancing carbon reduction measures. However, the process of soil respiration is extremely complex, and soil CO_2_ flux is influenced by numerous interacting factors [4,5,6]. It is strongly influenced by environmental variables such as soil temperature, moisture [7], and organic matter content. The flux is also affected by anthropogenic activities such as farming, fertilization [8,9], and other agricultural activities. The combined effects of these factors pose significant challenges for the accurate monitoring of soil CO_2_ flux.

The conventional methods for monitoring soil respiration primarily rely on the static chambers, dynamic chamber, and the micrometeorological methods [10,11]. The static chamber method is constrained by its relatively low measurement accuracy and its inability to perform continuous measurement. The dynamic chamber method, such as the LI-8100A automated soil CO_2_ flux system, offers advantages of high measurement accuracy and rapid response time. But this method also suffers from limitations including high cost [12], long preheating time, and limited portability [13,14]. Furthermore, the microclimate method requires specific conditions, such as soil surface homogeneity, suitable topography, and relatively stable environmental conditions [15,16], which restricts its measurement accuracy and practical applicability. These methods all exhibit varying degrees of portability, continuous monitoring capability, and cost-effectiveness. In addition, soil respiration exhibits significant spatial heterogeneity [17,18], complicating accurate and representative measurement.

Sensor technology is an emerging approach for gas detection. It offers significant advantages over traditional analytical instruments, including lower cost, improved portability, and reduced power consumption. Its detection reliability has been validated by multiple studies [19,20,21,22]. With the continuous and rapid advancement of low-cost sensing technologies, CO_2_ sensors based on non-dispersive infrared (NDIR) principles have been widely applied in carbon dioxide monitoring across diverse scenarios [19,23,24]. Early studies demonstrated that specialized NDIR sensors could be deployed for soil profile CO_2_ monitoring [25,26]. More recently, industrial-grade low-cost NDIR modules have been applied widely [27,28,29,30,31]. However, most systems lack the time synchronization mechanism between nodes and the remote wireless data transmission capability, failing to meet the requirements for synchronous multi-point monitoring.

To achieve the multi-point synchronous monitoring of soil CO_2_, wireless sensor networks have been applied. Common technologies include Wi-Fi, Bluetooth, and Long-range radio (LoRa). Among them, LoRa has the ability to remotely transmit data. Due to its use of spread spectrum modulation mechanism, it can maintain a stable path even in complex environments and under physical obstructions, demonstrating strong anti-interference capability [32]. Existing research has indicated that the transmission range of LoRa can transmit signals over a range of 5 to 10 km between urban buildings [33], confirming its technical feasibility for long-distance communication. Meanwhile, re-searchers have also developed a batch of IoT-connected soil CO_2_ sensor probes based on LoRa and conducted measurements within a 20 m cross section [34]. Although these probes are equipped with an independent real-time clock to add timestamps for local data, there is a lack of synchronization mechanism between the nodes. In addition, the reliability of this technology in monitoring large-scale and complex environments in the field still needs to be further verified. In addition, the time synchronization accuracy requirement for multi-point monitoring is a key issue [35], as even minor time deviations can lead to errors in flux calculations and compromise data reliability. Research indicates that the synchronization time accuracy for low-cost sensor system (LC-SS) [36] and SRS monitoring ranges from milliseconds to seconds, while the LI-8150 multiplexer [37] achieves accuracy of <100 ms. Therefore, there is an urgent need to develop monitoring equipment that is low-cost, high-precision, capable of wireless data transmission, and suitable for large-scale, multi-point synchronous monitoring while maintaining high portability.

This study aims to develop a novel, portable, and low-cost Soil Respiration System (SRS) based on a NDIR CO_2_ sensor, to address the bottlenecks posed by high time synchronization accuracy and the anti-interference wireless data transmission in large-scale field observations [38]. Through the design of synchronous parallel observation experiments of SRS and a reference instrument in homogeneous soil and field environments, and by comprehensive application using statistical methods, the reliability of the SRS was systematically validated. The study not only assessed the consistency of the SRS with the reference instrument but also comprehensively examined its monitoring stability under varying environmental conditions. It provides experimental validation and demonstration applications for the advancement of the low-cost, high-efficiency soil respiration monitoring technology.

## 2. Materials and Methods

### 2.1. Sensor Description

The SRS integrates a CO_2_ sensor (K33, SenseAir, Delsbo, Sweden) and a LoRa communication module. The CO_2_ sensor operates based on the principle of NDIR spectroscopy. It has a guaranteed detection range of 0–10,000 ppm, and can simultaneously monitor the temperature and relative humidity in the gas chamber. Its measurement interval can be user-configured, ranging from 30 s to 0.5 years. The sensor operates at a supply voltage of 4.5–14.0 V DC, with low power consumption. Detailed technical specifications are available on the manufacturer’s official website. The sensor is installed inside the soil respiration chamber, as shown in the Supplementary Materials (Figure S2).

### 2.2. Device Design

The SRS consists of four components: a soil ring, a soil respiration chamber, a data acquisition system, and a power supply system (Figure S1). (1) The soil ring is made of circular PVC pipe. During installation, the bottom of the ring is inserted into the soil to secure the soil respiration chamber and form a closed monitoring space. Inserting it into the soil in advance can minimize soil disturbance caused by the subsequent placement of the chamber. (2) The soil respiration chamber is a cylindrical hollow structure with an internal diameter of 20.2 cm and a height of 25.0 cm. It is sealed at the top and open at the bottom, and is integrated with a CO_2_ sensor inside. The top of each soil respiration chamber is independently equipped with a lithium battery and a LoRa communication module, and the external enclosure is opaque and waterproof. The side of the shell is equipped with a power switch and a charging interface, and a rubber rod antenna is mounted on the top. (3) The data acquisition system comprises a suction cup antenna, a LoRa main controller and a data logger (CR1000X, Campbell Scientific, Logan, UT, USA). (4) The power supply system uses a 12V lead-acid battery to directly power the data acquisition system.

**Figure 1:**
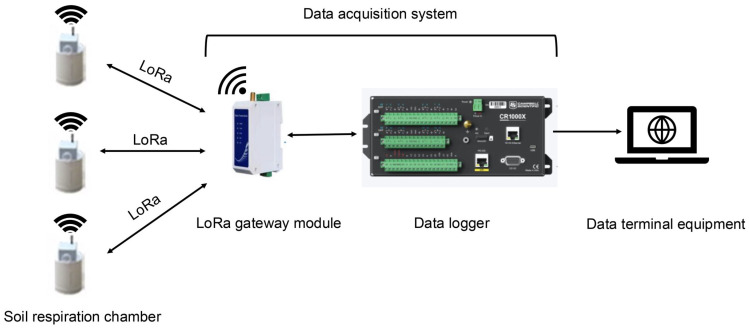
The workflow of the soil respiration system.

The SRS includes multiple soil respiration chambers, each housing an integrated CO_2_ sensor and a LoRa communication module. CO_2_ released by soil respiration accumulates within the chambers, where it is continuously monitored and detected by the internal sensors. All chambers transmit commands and receive data through a centralized CR1000X data logger (Campbell Scientific, Logan, UT, USA) and a LoRa gateway module (Figure 1). As all sensors are controlled by the same CR1000X, their measurement timestamps are synchronized by the unified time source of CR1000X. This fundamentally ensures temporal synchronization accuracy across all measurement points, enabling synchronous measurements of soil CO_2_ concentration across multiple time periods and locations. The data acquisition system centered on the CR1000X, employing LoggerNet 4.7 software for programing and operational control, and incorporates a LoRa communication module to enable remote data transmission. Detailed technical parameters, model specifications, and functional descriptions of all SRS components are provided in Table S2.

A single LoRa gateway theoretically supports 200 nodes [39], which means it can support 200 SRS units simultaneously at multiple locations. In reality, the number of SRS units are designed and deployed based on the dimensions of your study area. The SRS is characterized by high portability, low power consumption, and strong resistance to interference. It enables continuous soil CO_2_ monitoring and remote data transmission, making it suitable for multi-point and large-scale field applications.

### 2.3. Sensor Calibration and Performance Testing

**Figure 2:**
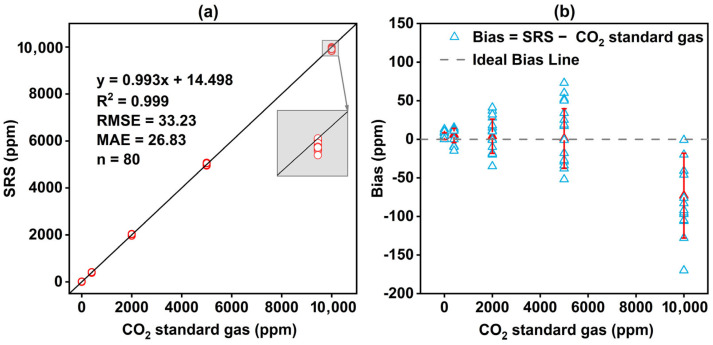
(**a**) Comparison between the SRS measurements with CO_2_ standard gas concentrations in span calibration: calibration result of the SRS is y = 0.993x + 14.498 (R^2^ = 0.999); (**b**) Bias (SRS—CO_2_ standard gas) as a function of CO_2_ standard gas concentration. The red line represents the error bars (standard deviation of the bias).

According to the sensor technical manual and established research practices [40], this study employed the zero-point calibration method for sensor calibration. During calibration, the 16 sensors were placed in a sealed chamber and continuously purged with high-purity nitrogen gas (CO_2_ concentration of 0 ppm). During this step, the Din2 terminal was short-circuited to the ground line and maintained for at least 8 s, to trigger the sensor’s zero-point calibration procedure. This process allows the sensor’s internal algorithm to adjust continuously and correct for zero-point drift. After completing zero calibration, span calibration followed using CO_2_ standard gases at 0 ppm, 400 ppm (ambient back-ground concentration), 2000 ppm, 5000 ppm, and 10,000 ppm. All gases had a standard uncertainty of ±1% and were introduced into the sealed calibration chamber at a stable flow rate of 1 L/min via pressure reducers and flow meters. Each sensor was continuously exposed for 5–10 min to ensure adequate contact and stabilization [41]. All calibration procedures were conducted under indoor constant temperature conditions (25 °C). The CO_2_ sensor in the SRS operates on a linear signal–concentration relationship. Because this linear response matches the sensor’s working principle, a linear calibration model was applied. Results confirmed a strong linear fit between SRS readings and standard gas values, yielding the equation y = 0.993x + 14.498 (R^2^ = 0.999) and root mean square error (RMSE) of 33.23 ppm (Figure 2a). At concentrations other than zero (400 to 10,000 ppm), the sensor exhibited excellent stability, with a coefficient of variation of 0.81%. Figure 2b displays the distribution of calibrated measurement deviations, with the standard deviations of bias ranging from 4.38 to 54.98 ppm. At the upper limit of the measurement range (10,000 ppm), the measurement deviations showed a negative distortion of the concentration. This phenomenon is due to saturation of the reaction near the upper limit of the sensors, resulting in a systematic underestimation of the actual concentrations.

Besides the initial full calibration at the start of the experiment, a zero calibration was performed for rapid verification before each monitoring round. Readings that deviated by more than ±30 ppm would have required re-calibration, but no re-calibration was needed during the entire study period.

To comprehensively evaluate the wireless communication performance of the SRS, including its response speed, communication range, data transmission success rate, and environmental adaptability, systematic testing were conducted in a range of representative environments. The test scenarios included both homogeneous environments and more complex environments with obstacles, such as open terrain and forested areas, to assess communication performance under diverse topographical and shading conditions. Furthermore, tests were carried out under seasonal climate conditions, specifically high summer temperatures and low winter temperatures, to examine the influence of temperature variations on data accuracy. To evaluate SRS communication reliability, we measured data reception success rates across varying transmission distances under these environmental and climatic conditions. These measurements support comprehensive assessment of the system performance in both simulated and real-world field applications.

### 2.4. Simulation Comparative Experiment

**Figure 3:**
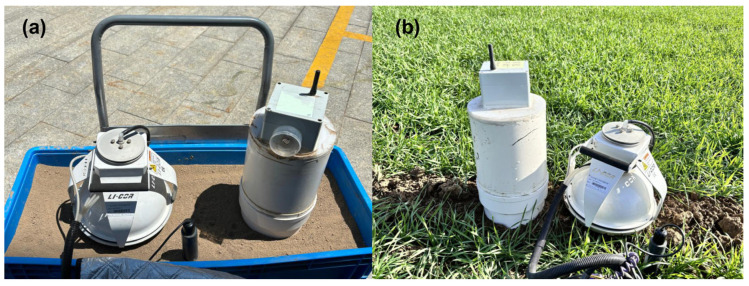
(**a**) Simulation comparative experiments conducted synchronously in homogeneous soil; (**b**) field comparative experiments were conducted in the wheat field.

To preliminarily validate the performance of the SRS, this study conducted a simulation comparative experiment under uniform soil conditions. The experiment utilized a PVC container (100 × 40 × 28 cm) filled with homogenized soil sieved through a 2 mm mesh. By eliminating potential interfering factors such as soil spatial heterogeneity and vegetation cover, a standardized testing environment was established (Figure 3a). This experimental design minimized the influence of complex environmental variables, ensuring physical properties of the soil measured by both instruments. This approach also enabled an accurate evaluation of the system’s performance. The experiments were conducted during winter 2024, spring 2025, and summer 2025. Each experiment began at 06:00 and continued until 18:00 to evaluate the stability of the system’s responses under different seasonal environments.

Prior to each experiment, the SRS and LI-8100A soil rings were installed side-by-side at least 24 h in advance. They were inserted into the soil base-down, leaving the collar rims extending approximately 3 cm above the surface. Measurements began once the soil conditions had equilibrated. For data collection, the chambers of both systems were positioned on their respective collars. A gap of 10–20 cm was maintained between the outer edges of the chambers to minimize cross-interference. Following each monitoring cycle, both chambers were raised together. The next measurement cycle commenced after a 30 min delay. Throughout the CO_2_ measurement periods, we simultaneously monitored soil temperature and volumetric moisture content. These parameters were recorded using a soil three-parameter analyzer (TDR150, Spectrum Technologies, Aurora, IL, USA).

### 2.5. Field Comparative Experiment

**Figure 4:**
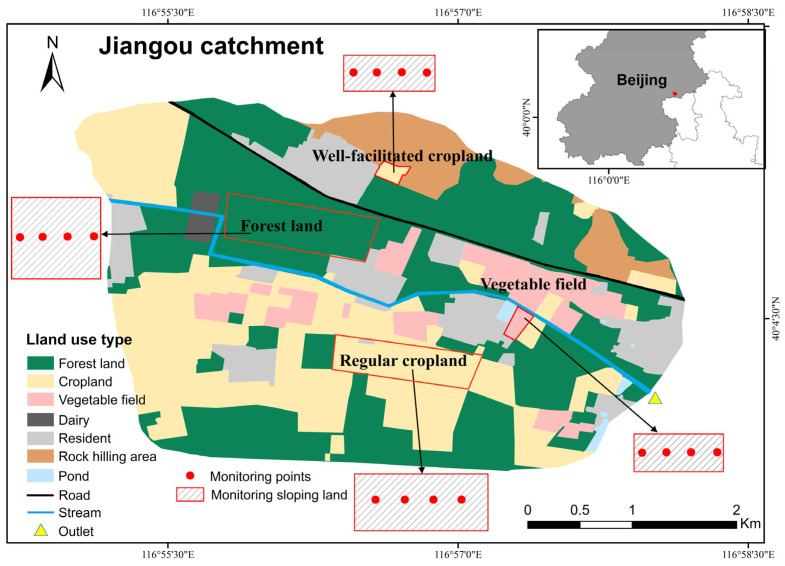
Location of the Jiangou catchment with spatial distribution of monitoring sloping lands in field comparative experiments of soil respiration.

The field comparative experiments aim to verify the reliability and ecological applicability of the SRS under real environmental conditions. This study conducted synchronous comparative observations using the SRS and LI-8100A across different land use types and seasonal conditions within the catchment (Figure 3b), allowing the systematic evaluation of the SRS measurement performance under various ecological environments and climatic conditions. The field observation site was located in the Jiangou agricultural catchment (40°48′00″ N, 116°56′30″ E), covering an area of approximately 14 km^2^ in Beijing, China (Figure 4). The region is characterized by a warm temperate semi-humid monsoon climate, with a mean annual temperature of 11.8 °C and a long-term average annual rainfall of 610 mm. The primary land use types in the catchment were forest land (40.19%), cropland (31.69%, including regular cropland and well-facilitated cropland), vegetable fields (5.97%), residences (13%), rock hilly area (7%) and so on. Forest land mainly consists of mature White Poplar trees with low density. Cropland follows a winter wheat–summer corn rotation system, while vegetable fields employ an open-field cultivation model (primarily growing Chinese cabbage, cabbage, radishes, etc.). The laboratory analysis results indicated that the soil organic carbon content of forest land (12.64 ± 2.73 g/kg) was the highest, followed by well-facilitated cropland (8.74 ± 0.54 g/kg) and vegetable fields (5.88 ± 0.77 g/kg), with regular cropland (4.38 ± 0.61 g/kg) being the lowest. In terms of soil mechanical composition, all land use types exhibited a sand-dominated particle size (0.02–2 mm), accounting for 43.33% to 59.93% of the total composition (Table S1).

The reference instrument (LI-8100A, Li-Cor Inc., Lincoln, NE, USA) is a single-point measurement device, it proved difficult to establish paired comparative monitoring synchronously for all 75 SRS units during the field comparison experiments, which is based on the area of our study catchment. To ensure the completement of the comparative monitoring for all land use types within one day, only 16 different chambers were used for synchronous validation with the LI-8100A during field comparative experiments. The field comparative experiments were conducted in the above land use types during the winter of 2024 and summer of 2025, where one representative hillslope for each land use type was selected. On each slope, four monitoring points were uniformly established from upper to lower elevations along a transect. Slope lengths varied across different land use types, with cropland and forest land slopes of 260 m and 100 m in length, respectively, while vegetable fields (~50 m) featured shorter slopes. Adjacent monitoring points were spaced 20 to 60 m apart. This range was determined by the actual slope length of each site to demonstrate the performance of synchronous remote data transmission. To minimize soil disturbance from instrument installation, soil rings were embedded in advance into the bare ground. This study conducted daytime monitoring of soil CO_2_ fluxes across different land use types in December and June. During the field comparative experiment, different land types exhibited distinct agricultural phases and vegetation conditions: In December, cropland (Packet Reception Ratio, PRR: 98%) was in the wheat emergence stage, while vegetable fields (PRR: 98%) were fallow. By June, cropland entered the wheat harvest period, and vegetable fields were in the cabbage growth phase. Forest land (PRR: 95%) consisted of low-density mature forest, maintaining relatively stable vegetation cover and shading levels throughout the year. In forest land, if plants were growing within the base, they were trimmed flush with the ground and disturbing of the surface litter was avoided as much as possible. Prior to taking measurements, the bottom of each soil collar was inserted into the ground, leaving the top rim approximately 3 cm above the surface. To ensure spatial consistency in measurement conditions for both instruments, the SRS and LI-8100A gas chambers were positioned side-by-side on their respective soil rings prior to measurement. A 10–20 cm spacing was maintained between their edges to adjacent chambers. This spacing also ensured that both chambers monitor a highly consistent soil microenvironment. Subsequently, both systems were activated simultaneously to conduct synchronized monitoring. Soil temperature and moisture content were recorded concurrently throughout the experiment. Since the LI-8100A is a single-point monitor, to ensure temporal comparability between SRS and LI-8100A measurements of soil CO_2_ flux, we selected values collected at the same time point for comparison. Due to delays in the installation and debugging of the LI-8100A, the monitoring periods of the two field campaigns were not fully synchronized.

### 2.6. Data Analysis

The SRS measures CO_2_ concentration directly (unit: ppm). The CO_2_ flux (F) is calculated using the following equation:

(1) $$F\;=\;\rho \;\times H\times \frac{△C}{△t}\;\times \;\frac{273}{273+T}\times \frac{1000}{44}$$

where *F* represents the CO_2_ flux (μmol·m^−2^·s^−1^), *ρ* denotes the density of CO_2_ under standard conditions (1.977 kg·m^−3^), *H* is the height of the monitoring (m) chamber, $\frac{△C}{△t}$ indicates the rate of change in CO_2_ concentration inside the chamber per unit time (s), and *T* is the temperature inside the chamber during monitoring (°C). The coefficient $\frac{1000}{44}$ is used for unit conversion: 44 represents the molar mass of CO_2_ (g·mol^−1^), enabling the conversion from mass (mg) to amount of substance (μmol).

This study utilized SPSS 22.0 (SPSS Inc., Chicago, IL, USA) for correlation analysis and significance testing of the monitoring from the two instruments. Based on the Origin 2022 (OriginLab Corp., Wellesley Hills, MA, USA) software platform, linear regression analysis and Bland–Altman analysis were performed on the monitoring results from both systems to comprehensively evaluate the accuracy and reliability of the SRS in practical monitoring. Microsoft Excel 2021 (Microsoft Corp., Redmond, WA, USA) was used to calculate the RMSE between the monitoring of the two systems, thereby quantifying the overall monitoring error of the SRS.

## 3. Results

### 3.1. Simulation Comparative Experiments for Soil CO_2_ Monitoring

**Figure 5:**
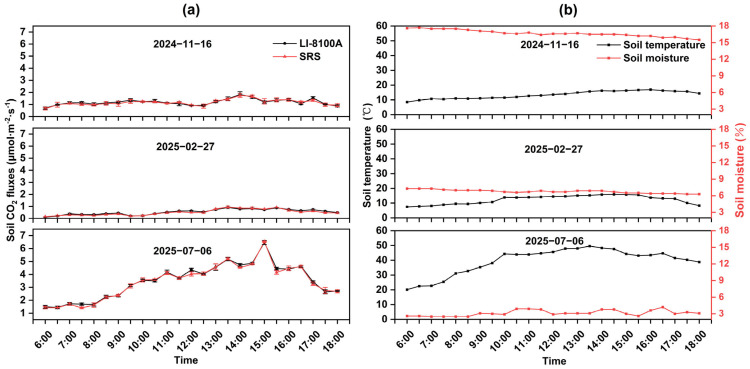
(**a**) Temporal variation in soil CO_2_ flux measured by the SRS and the LI-8100A during the simulation comparative experiments. (**b**) Temporal variations in soil temperature and moisture content during the simulation comparative experiments.

Based on observational data from the simulation comparative experiments, soil CO_2_ flux exhibited pronounced seasonal variation patterns (Figure 5a). Statistical analysis revealed significant differences in soil CO_2_ flux among the three experimental periods (*p* < 0.001), with lowest fluxes in February 2025 and similar values of the SRS (0.51 μmol·m^−2^·s^−1^) and LI-8100A (0.54 μmol·m^−2^·s^−1^). Intermediate levels were observed in November 2024 (1.16 and 1.19 μmol·m^−2^·s^−1^). The highest flux levels were recorded in July 2025, reaching values of 3.40 μmol·m^−2^·s^−1^ for the SRS and 3.57 μmol·m^−2^·s^−1^ for the LI-8100A. Across all seasons, the measurement from the SRS and LI-8100A remained highly consistent. The flux ranges recorded by the two systems largely overlapped, and the differences in their mean values remained consistently below 0.20 μmol·m^−2^·s^−1^. From the perspective of the daily variation, the measured values in February and November fluctuated relatively gently between days. Peak fluxes occurred at 13:30 in February (0.96 and 0.91 μmol·m^−2^·s^−1^ for the SRS and LI-8100A, respectively) and at 14:00 in November (1.75 and 1.82 μmol·m^−2^·s^−1^, respectively). In contrast, the July measurements displayed a pronounced unimodal pattern, increasing with rising soil temperature, peaking around 15:00 (6.53 μmol·m^−2^·s^−1^ for SRS and 6.47 μmol·m^−2^·s^−1^ for LI-8100A), and subsequently diminishing.

The corresponding soil temperature and moisture dynamics are shown in Figure 5b. During the monitoring period, the average soil temperatures in February and November were similar (12.3 °C and 13.5 °C, respectively), while the average temperature in July was substantially higher (39.5 °C), peaking around 13:30. The average soil moisture content also showed marked differences, with November (16.7%) being substantially higher than February (6.8%) and July (3.1%).

**Figure 6:**
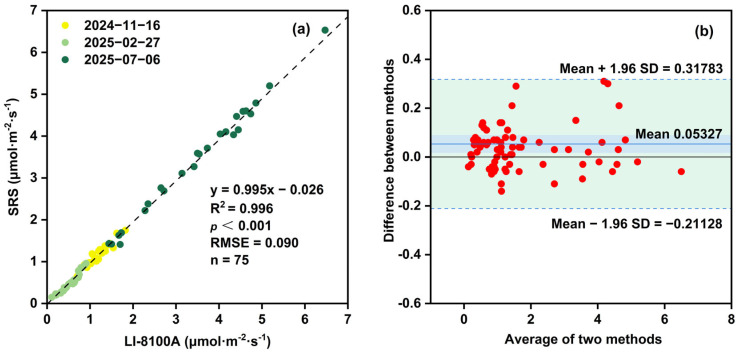
(**a**) Linear regression between soil CO_2_ flux measurement the SRS and LI-8100A in the simulation comparative experiments. (**b**) Bland–Altman analysis of the soil CO_2_ measurements from the simulation comparative experiments. The green area represents the range between the two horizontal dashed lines (Mean + 1.96 SD and Mean − 1.96 SD) in the figure, denoting the interval covered by the 95% confidence interval. The solid blue line (Mean = 0.05327) indicates the average “difference between methods” across all measurement points, reflecting the systematic bias between the two measurement methods.

Linear regression analysis between measurements obtained from the SRS and LI-8100A yielded the equation y = 0.995x − 0.026, with a high coefficients of determination (R^2^) value of 0.996 and statistical significance of *p* < 0.001 (Figure 6a), indicating an extremely strong positive linear correlation between the two systems. The RMSE of 0.090 μmol·m^−2^·s^−1^ further demonstrates the high accuracy of the SRS under practical monitoring conditions.

To further assess the agreement between the two systems, Bland–Altman analysis was conducted (Figure 6b). Approximately 95% of the data points fell within the limits of agreement, ranging from −0.211 to 0.318 μmol·m^−2^·s^−1^. The narrow limits of agreement, combined with a mean difference close to zero, indicates excellent consistency between the SRS and LI-8100A.

### 3.2. Field Comparative Experiments for Soil CO_2_ Monitoring

**Figure 7:**
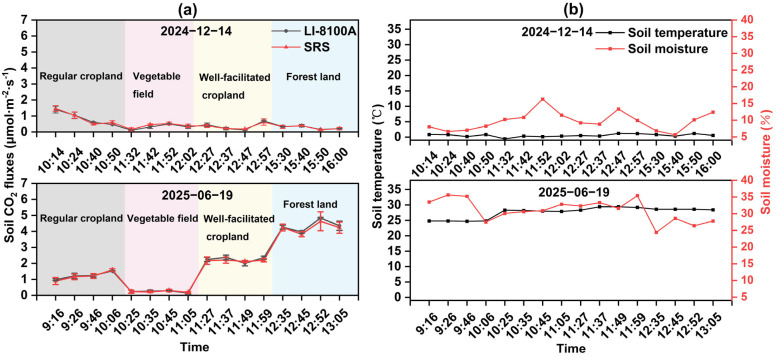
(**a**) Trend of soil CO_2_ fluxes measured by soil respiration system and LI-8100A over time in field comparative experiments. (**b**) The temporal variations in soil temperature and moisture content in two field comparative experiments.

In the field comparative experiments, the temporal variations in soil CO_2_ flux measured by the SRS and the LI-8100A were highly consistent (Figure 7a). In December 2024, the mean soil CO_2_ fluxes measured by the two systems were 0.48 and 0.47 μmol·m^−2^·s^−1^, respectively, with smooth diurnal fluctuations. By June 2025, with rising temperatures, the mean fluxes rose to 1.96 for the SRS and 2.02 μmol·m^−2^·s^−1^ for the LI-8100A, exhibiting pronounced diurnal variation. Peak values were recorded in the afternoon, reaching 4.63 μmol·m^−2^·s^−1^ for the SRS and 4.83 μmol·m^−2^·s^−1^ for the LI-8100A. Statistical analysis confirmed that soil CO_2_ flux in June was significantly higher than in December (*p* < 0.001).

The corresponding soil temperature and moisture dynamics are shown in Figure 7b. In December 2024, soil temperature exhibited minimal diurnal variation, with a mean value of 0.57 °C, while moisture content varied substantially among land use types, with the lowest value observed in regular cropland (7.45%) and the highest in vegetable fields (12.43%). By June 2025, soil temperature increased markedly to a mean value of 27.69 °C and exhibited clear diurnal variation, peaking at midday (29.2 °C). The corresponding moisture content followed the order: forest land (26.47%) < vegetable fields (31.43%) < regular cropland (32.2%) < well-facilitated cropland (33.43%).

**Figure 8:**
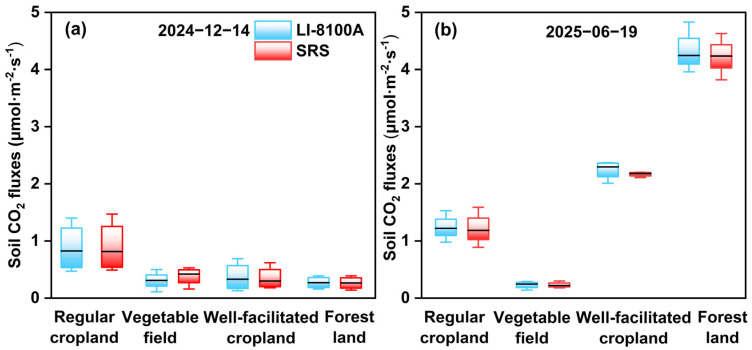
Distribution of soil CO_2_ flux measured simultaneously by the SRS and LI-8100A across different land use types: (**a**) 14 December 2024; (**b**) 19 June 2025. The horizontal line indicates the median.

The soil CO_2_ fluxes results of the SRS and LI-8100A across different land use types are shown in Figure 8. In December, both instruments recorded the highest mean soil CO_2_ flux in regular cropland (0.90 ± 0.45 μmol·m^−2^·s^−1^ for SRS and 0.88 ± 0.43 μmol·m^−2^·s^−1^ for LI-8100A), while forest land exhibited the lowest values (0.27 ± 0.11 μmol·m^−2^·s^−1^ for SRS and 0.27 ± 0.11 μmol·m^−2^·s^−1^ for LI-8100A). In the following June, however, both systems measured the highest flux in forest land (4.23 ± 0.33 μmol·m^−2^·s^−1^ for SRS and 4.35 ± 0.36 μmol·m^−2^·s^−1^ for LI-8100A) and the lowest in vegetable fields (0.23 ± 0.05 μmol·m^−2^·s^−1^ for SRS and 0.23 ± 0.06 μmol·m^−2^·s^−1^ for LI-8100A).

**Figure 9:**
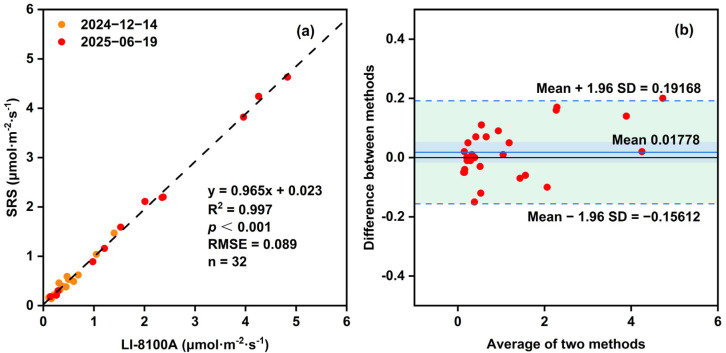
(**a**) Linear regression between soil CO_2_ flux measurement the SRS and LI-8100A in the field comparative experiments. (**b**) Bland–Altman analysis of the measured values of the field comparative experiments. The green area represents the range between the two horizontal dashed lines (Mean + 1.96 SD and Mean − 1.96 SD) in the figure, denoting the interval covered by the 95% confidence interval. The solid blue line (Mean = 0.01778) indicates the average “difference between methods” across all measurement points, reflecting the systematic bias between the two measurement methods.

Linear regression analysis of the experimental measurements from the experiments yielded the equation y = 0.965x + 0.023, with a high coefficient of determination (R^2^ of 0.997) and a strong statistical significance (*p* < 0.001) (Figure 9a). These results indicate a nearly negligible systematic deviation and a highly consistent response relationship between the SRS and LI-8100A. The RMSE of 0.089 μmol·m^−2^·s^−1^ further confirms the high measurement accuracy of the SRS under field comparative conditions.

Bland–Altman analysis provided additional quantitative evidence of agreement between the two instruments (Figure 9b), with most data points falling within the limits of the agreement (LoA: −0.156 to 0.192 μmol·m^−2^·s^−1^), with a mean difference approaching zero.

## 4. Discussion

### 4.1. Soil CO_2_ Flux Dynamics

The soil CO_2_ flux ranges observed in both the simulated and field comparative experiments in this study are consistent with those reported in previous research, indicating the reliable monitoring capability of the SRS. In the February simulation comparative experiment, the mean soil CO_2_ flux measured by the SRS was 0.52 μmol m^−2^ s^−1^, which closely matched the winter bare-soil CO_2_ flux of approximately 0.50 μmol m^−2^ s^−1^ reported by Li et al. [42]. Similarly, the soil CO_2_ flux ranges recorded by the SRS in regular and well-facilitated cropland are consistent with those reported by Mühlbachová et al. [43], who documented six-year average fluxes under conventional and reduced tillage conditions of 6.1 and 3.1 μmol m^−2^ s^−1^, respectively. Furthermore, the SRS successfully captured the seasonal dynamics of soil CO_2_ flux in forest land, with higher mean flux in summer (4.23 μmol·m^−2^·s^−1^) than in winter (0.27 μmol·m^−2^·s^−1^). The measured values were consistent with those in previous studies conducted in Beijing mountainous areas (0.12–4.87 μmol·m^−2^·s^−1^) [44].

Results from the simulation experiments showed that both the mean and peak values of soil CO_2_ flux in summer are significantly higher than those observed in winter, indicating a strong seasonal contrast. The summer–winter comparison highlights that temperature is a key environmental driver of soil respiration dynamics [45,46]. Field comparative experiments further confirmed this pattern. Soil CO_2_ fluxes across all land use types increased progressively from lower levels in December to higher values by the following June, in parallel with rising temperatures and the advancement of the growing season [47,48].

During winter, due to lower ambient temperatures and higher soil moisture content in well-facilitated cropland, partial soil freezing occurred [49] and this suppressed microbial respiration. Consequently, the CO_2_ flux in well-facilitated cropland was lower than that in regular cropland during this period. In summer, measurement in well-facilitated cropland was conducted at midday when soil temperatures were relatively high, whereas measurements in regular cropland were taken in the morning when soil temperatures were lower. Furthermore, well-facilitated cropland is equipped with more advanced irrigation facilities, maintaining soil moisture within a range optimal for soil respiration. Its higher soil fertility also effectively promotes crop root growth and microbial activity [50], collectively enhancing soil CO_2_ emissions. As a result, the soil CO_2_ flux in well-facilitated cropland was significantly higher than in regular cropland. Forest land exhibited the highest soil CO_2_ flux in summer, likely due to the stable soil structure, favorable aeration conditions, and continuous organic carbon input from persistent leaf litter [51,52]. In contrast, vegetable fields consistently showed the lowest soil CO_2_ fluxes in both December and June. This pattern may be attributed to site-specific management practices, where frequent irrigation leads to high soil moisture levels that suppress aerobic microbial activity [53,54]. In addition, short-cycle crop rotation disturbs soil structure and further limits carbon input and reduces soil respiration [55].

### 4.2. Performance of the SRS

This study developed a low-cost, high-precision, and portable soil CO_2_ monitoring system (SRS). The SRS supports large-scale deployment and remote data transmission. It achieves a communication range of up to 2 km in field environments. Through both simulation and field comparative experiments, the system’s functionality and overall advantages were validated.

In terms of technical performance, the NDIR sensor employed in the SRS exhibits a response time of less than 25 s, outperforming the MHZ19B sensor reported by Bonilla et al. [56], which has a response time exceeding 60 s. The SRS achieves a Packet Reception Ratio (PRR) of 98% in open terrain without obstructions (exceeding 99% in laboratory environments), while LoRa can transmit data between urban buildings at distances of 5 to 10 km [57]. This study tested SRS in a wooded environment with a 2 km transmission distance and a 95% packet reception rate, meeting or exceeding the performance of comparable CO_2_ monitoring systems. For example, Jiang reported a PRR of 99.87% [58], while Khan et al. documented a PRR exceeding 97% for an ESP-MESH-based system [59]. In addition, the SRS features low power consumption and can operate continuously for approximately 14 days, demonstrating superior battery endurance compared to the LI-8100A.

In terms of measurement accuracy, this study validated the SRS using CO_2_ standard gas, revealing a mean absolute error (MAE) of 26.83 ppm between the SRS measurements and the reference values (Figure 2a). This value falls below the factory-specified nominal accuracy of ±30 ppm. The SRS and LI-8100A exhibited highly synchronized temporal trends (Figure 5a and Figure 7a), with the mean difference consistently below 0.20 μmol·m^−2^·s^−1^. A high correlation (R^2^ > 0.99) and a low root mean square error (RMSE ≤ 0.090 μmol·m^−2^·s^−1^) were observed between the SRS and LI-8100A, indicating comparable performance. Similar results have been reported for other low-cost monitoring systems, e.g., the K33SOIL CO_2_ probe exhibited an RMSE of 0.032% CO_2_ when compared with LI-6400 and Vaisala GMP343 [29], and the LC-SS demonstrated an RMSE of 0.15 μmol·m^−2^·s^−1^ relative to the LI-8100A [36].

The observed agreement primarily reflects the inherent reliability of the NDIR sensor, while minor deviations may be attributed to the combined effects of the sensor’s inherent accuracy limits and microenvironmental variations among different measurement chambers.

### 4.3. Strengths and Limitations

Compared with other commercial instruments, the SRS demonstrates clear advantages in both cost-effectiveness and overall performance (Table 1). Neither the LI-8100A nor the K33SOIL support multi-point synchronous monitoring. While the LI-8150 does supports this functionality, it is considerably more expensive. The cost of the SRS is approximately $820, representing a reduction of about 97.9% compared to the LI-8100A (approximately $40,000) and a reduction of about 99.4% compared to the LI-8150 (approximately $142,000). The SRS uses the same CR1000X data logger for time stamping, ensuring highly accurate time synchronization (millisecond to second) between different monitoring points. Although the time synchronization accuracy of LC-SS is millisecond to second, it only supports synchronized observations at 6 points, which limits its monitoring capabilities (Table 1). In contrast, the SRS is not only low-cost and portable but it also integrates LoRa wireless transmission capabilities, enabling rapid redeployment across different sampling locations. Its low power consumption and high portability further enhance its practicality for large-scale, multi-point synchronous monitoring in the field. These features effectively address the limitations of conventional instruments in terms of operational efficiency and spatial coverage [29,60]. The system thereby provides an economical and reliable technical solution for capturing the spatiotemporal heterogeneity of soil CO_2_ fluxes. The above advantages make the SRS a practical choice for large-scale, multi-point synchronous monitoring of soil CO_2_. In addition, the SRS requires pre-embedding soil rings at least 2 h in advance to allow stabilization of the soil microenvironment for short-term measurements, whereas long-term continuous monitoring requires only a single pre-installation at the beginning of the experiment. Our low-cost system provides a balanced pathway for soil management transformation that reconciles farmers’ livelihoods with climate goals, aligns with carbon-nitrogen cycle research, and informs climate mitigation strategies [61].

Although the SRS demonstrated good consistency in both simulation and field comparative experiments, the application scenarios of SRS and the validation methods employed in this study still have certain limitations. First, the SRS is limited by its sample size. It may cause an overfitting problem, but we performed 75 paired sets of the SRS and LI-8100A data (*p* < 0.001) as the independent spatial validation. Secondly, the calibration model requires optimization using larger datasets. Future work could consider integrating environmental data such as temperature and humidity, and incorporating machine learning to construct calibration models. Additionally, the SRS is not yet suitable for monitoring in wetlands, marshes, or submerged conditions. Future research should conduct systematic validation across broader soil textures, ecosystem types, and climatic regions. It should also extend the environmental adaptability of the monitoring system to multi-medium environments such as aquatic systems and wetlands, enabling large-scale synchronous monitoring of CO_2_ fluxes across different ecosystems.

## 5. Conclusions

This study successfully developed a low-cost, portable soil respiration system (SRS) utilizing NDIR sensor and LoRa wireless communication, which has been applied to the monitoring of soil CO_2_ fluxes across multiple land use types at the catchment scale. The experimental results demonstrated the high reliability of the SRS, showing strong agreement with the reference instrument LI-8100A, with R^2^ > 0.99 in simulated and field comparative experiments. Bland–Altman analysis further confirmed measurement consistency, with over 95% of data points falling within the limits of agreement. The self-developed SRS not only substantially reduced cost, but it also maintained high measurement accuracy, supporting multi-point synchronous monitoring, and enabling remote data transmission. These features presented an efficient and practical solution for capturing spatial heterogeneity of soil respiration and showed great potential for broad application in carbon flux research. Future research will focus on developing chamber structures suitable for aquatic environments to further enhance the system’s environmental adaptability. In parallel, long-term continuous monitoring will be conducted to evaluate the system’s performance and its reliability in extended carbon flux network observations. Overall, the SRS offers a reliable technical solution for large-scale, multi-point soil carbon flux research.

## Figures and Tables

**Table 1:** Cost and performance comparison.

	SRS	LI-8100A Automated Soil CO_2_ Flux System	LI-8150 Multiplexer [37]	K33SOIL [29]	LC-SS [36]
Unit cost	$820	$40,000	Approximately $142,000	Low cost	$700
Portability	High	High	Low	High	High
Multi-point synchronous monitoring	Support	Not supported	Support	Not supported	Support
Number of synchronized monitoring points	75	1	16	1	6
Time synchronization accuracy	Millisecond to second	-	Millisecond	-	Millisecond to second
Wireless data transmission	Support (LoRa)	Not supported	Not supported	Not supported	Not supported
Power consumption	Low	High	High	Low	Low
Applicable scene	Single-point, multi-point, and large-scale monitoring	Single-point monitoring	Multi-point monitoring	Multi-point monitoring	Multi-point monitoring

## Data Availability

The raw data supporting the conclusions of this article will be made available by the authors on request.

## Supplementary Materials

The following supporting information can be downloaded at: https://www.mdpi.com/article/10.3390/s26030761/s1, Figure S1: The CO_2_ sensor used in the soil respiration system; Figure S2: The structural composition of the soil respiration system. In the picture, 1: Antenna; 2: Soil respiration air chamber; 3: Sensor; 4: Battery; 5: LoRa module; 6: Power switch; 7: Charging port; Table S1. Soil mechanical composition across different land use types. Table S2: The components of the soil respiration system. Table S3. In the field comparative experiments, the flux values, RMSE, coefficient of variation of each point position of SRS and LI-8100A.

## Abbreviations

The following abbreviations are used in this manuscript:

SRS	Soil Respiration System
NDIR	Non-dispersive infrared
LoRa	Long-range radio
RMSE	Root mean square error
MAE	Mean absolute error
R^2^	R-squared
PRR	Packet Reception Ratio
