# Supplementary material for: Application of a High-Performance, Low-Cost Portable NDIR Sensor Monitoring System for Continuous Measurements of In Situ Soil CO2 Fluxes
Source: Sensors (Basel). 2026 Jan 23;26(3):761. doi: 10.3390/s26030761 (PMC12899407; doi:10.3390/s26030761)
Supplement: Supplementary file 1 [file sensors-26-00761-s001.zip › sensors-4086224-supplementary.pdf]

Supplementary file

Application of a High-Performance, Low-Cost Portable NDIR Sensor Monitoring System for Continuous Measurements of In Situ Soil CO_2 Fluxes

Xinyuan Zeng [1], Xiaoyan Chen [1], Lee Heng [2], Suarau Odutola Oshunsanya [3] and Hanqing Yu [1,*]

1 Institute of Environment and Sustainable Development in Agriculture, Chinese Academy of Agricultural Sciences (CAAS), Haidian District, 100081 Beijing China.
2 International Atomic Energy Agency, A-1400 Vienna, Austria.
3 Department of Soil Resources Management, University of Ibadan, Ibadan 200005, Nigeria.
* Correspondence: yuhanqing@caas.cn (H. Yu), Tel & Fax: +86-10-82106016

Description of the soil respiratory system

Each soil respiration chamber (Figure S1) is equipped with an independent LoRa communication module operating at 433 MHz. During operation, the chambers can be accessed in either point-to-point or point-to-multipoint mode via a LoRa master controller. Each chamber is assigned a unique communication address, which is labeled on the sensor module inside the chamber, as shown in Figure S2. For example, in the label "A9", the trailing number indicates the communication address—in this case, "9".

The Soil Respiration System (SRS) primarily consists of four components: a soil collar, a soil respiration chamber, a data acquisition system, and a power supply system (Table S1). Each SRS unit is equipped with multiple soil respiration chambers, and each chamber integrates a CO_2 sensor and a LoRa communication module. All chambers are centrally managed through a unified CR1000X data logger and LoRa master controller, which together handle command transmission and data reception.

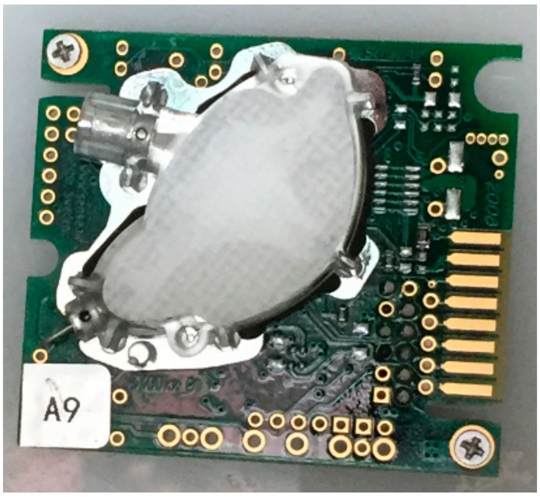

Figure S1. The CO_2 sensor used in the soil respiration system.

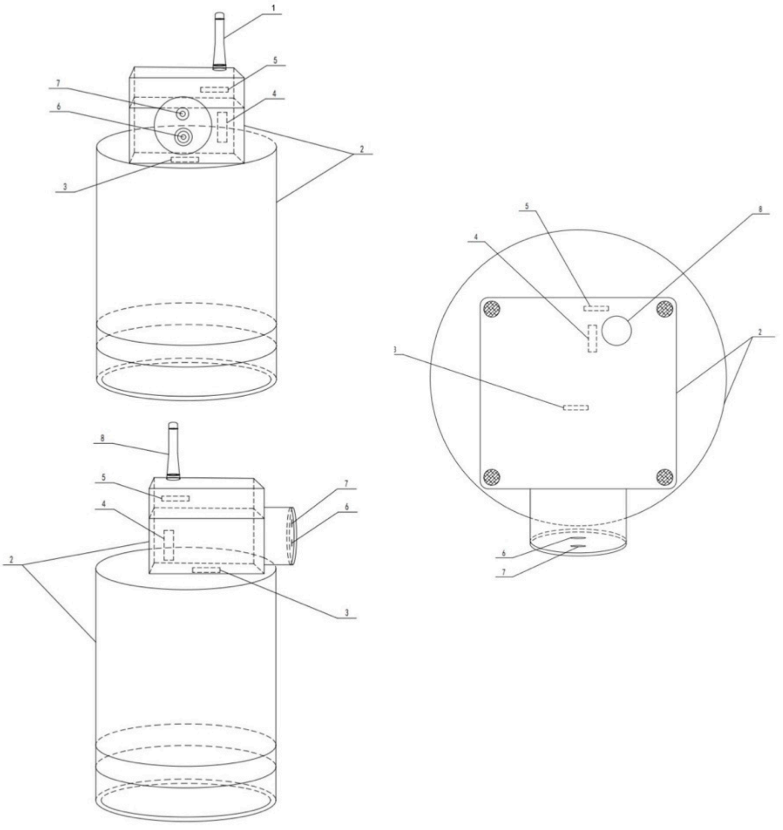

Figure S2. The structural composition of the soil respiration system. In the picture, 1: Antenna; 2: Soil respiration chamber; 3: Sensor; 4: Battery; 5: LoRa module; 6: Power switch; 7: Charging port.

Table S1. Soil mechanical composition across different land use types.

Land use types	Clay particles (<0.002 mm)/ %	Silt particles (0.002–0.02 mm)/ %	Sand particles (00.02–2 mm)/ %
Forest land	8.11	31.96	59.93
Regular cropland	12.85	41.75	45.40
Well-facilitated cropland	13.40	43.27	43.33
Vegetable fields	9.32	34.99	55.69

Table S2. The components of the soil respiration system.

Part	Specification	Purpose
Soil ring	PVC material, dimensions (inner diameter 20 cm, height 6 cm)	Fix the air chamber, construct a sealed measurement space, and pre-bury it to reduce soil disturbance
Soil respiration chamber	Dimensions: 20.2 cm (inner diameter) × 25 cm (height). Integrated components: CO_2 sensor, LoRa module	Monitoring soil CO_2 concentration, as well as chamber temperature and humidity
Data acquisition system	Including CR1000X datalogger and LoRa gateway module	Collect and store the data monitored by the sensors
Power supply system	12V lead-acid battery	Provide power support for the data acquisition system

Table S3. In the field comparative experiments, the flux values, RMSE, coefficient of variation of each point position of SRS and LI-8100A.

Land use types	Time	Flux values of SRS /μmol·m^{-2}·s^{-1}	Flux values of LI-8100A /μmol·m^{-2}·s^{-1}	RMSE	CV
Regular cropland (2024.12.14)	10:14	1.4	1.47	0.07	3.45%
	10:24	1.05	1.04	0.01	0.68%
	10:40	0.6	0.49	0.11	14.27%
	10:50	0.47	0.59	0.12	16.01%
Vegetable field (2024.12.14)	11:32	0.11	0.16	0.05	26.19%
	11:42	0.31	0.46	0.15	27.55%
	11:52	0.5	0.53	0.03	4.12%
	12:02	0.31	0.38	0.07	14.35%
Well-facilitated cropland (2024.12.14)	12:27	0.45	0.38	0.07	11.93%
	12:37	0.21	0.22	0.01	3.29%
	12:47	0.13	0.18	0.05	22.81%
	12:57	0.69	0.62	0.07	7.56%
Forest land (2024.12.14)	15:30	0.33	0.32	0.01	2.18%
	15:40	0.39	0.39	0	0.00%
	15:50	0.16	0.14	0.02	9.43%
	16:00	0.21	0.21	0	0.00%
Regular cropland (2025.6.19)	9:16	0.98	0.89	0.09	6.81%
	9:26	1.21	1.16	0.05	2.98%
	9:46	1.24	1.21	0.03	1.73%
	10:06	1.53	1.59	0.06	2.72%
Vegetable field (2025.6.19)	10:25	0.23	0.23	0	0.00%
	10:35	0.26	0.21	0.05	15.04%
	10:45	0.29	0.3	0.01	2.40%
	11:05	0.14	0.18	0.04	17.68%
Well-facilitated cropland (2025.6.19)	11:27	2.24	2.17	0.07	2.24%
	11:37	2.37	2.2	0.17	5.26%
	11:49	2.01	2.11	0.1	3.43%
	11:59	2.35	2.19	0.16	4.98%
Forest land (2025.6.19)	12:35	4.26	4.24	0.02	0.33%
	12:45	3.96	3.82	0.14	2.54%
	12:52	4.83	4.63	0.2	2.99%
	13:05	4.35	4.23	0.12	1.98%
